# Induction of arthritis by high mobility group box chromosomal protein 1 is independent of tumour necrosis factor signalling

**DOI:** 10.1186/ar2445

**Published:** 2008-06-26

**Authors:** Rille Pullerits, Ing-Marie Jonsson, George Kollias, Andrej Tarkowski

**Affiliations:** 1 Department of Rheumatology and Inflammation Research, Sahlgrenska Academy at Göteborg University, Guldhedsgatan 10A, 41346, Göteborg, Sweden; 2 Institute of Immunology, Biomedical Sciences Research Center 'Alexander Fleming', 34 Al. Fleming Street, 16672 Vari, Greece

## Abstract

**Introduction:**

TNFα and high mobility group box chromosomal protein 1 (HMGB1) are two potent proinflammatory cytokines implicated as important mediators of arthritis. Increased levels of these cytokines are found in the joints of rheumatoid arthritis patients, and the cytokines trigger arthritis when applied into the joints of naïve mice. HMGB1 is actively released from immune cells in response to TNFα; once released, HMGB1 in turn induces production of several proinflammatory cytokines – including IL-6 and TNFα – by macrophages. Whether HMGB1-induced arthritis is mediated via the TNFα pathway, however, is unknown. The purpose of the present study was to investigate whether the arthritis-inducing effect of HMGB1 is dependent on TNFα expression *in vivo *and to assess whether TNFα deficiency affects a proinflammatory cytokine response to HMGB1 *in vitro*.

**Methods:**

TNFα knockout mice and backcrossed control animals on a C57Bl6 background were injected intraarticularly with 5 μg HMGB1. Joints were dissected 3 days after intraarticular injection and were evaluated histologically by scoring the frequency and severity of arthritis. For *in vitro *studies, mouse spleen cultures from TNFα knockout mice and from control mice were incubated with different doses of HMGB1, and cell culture supernatants were collected at different time points for analysis of IL-6.

**Results:**

Intraarticular injection of HMGB1 into healthy mouse joints resulted in an overall frequency of 32% to 39% arthritic animals. No significant differences were found with respect to the severity and incidence of synovitis between mice deficient for TNFα (seven out of 18 mice with arthritis) in comparison with control TNFα^+/+ ^animals (six out of 19). No significant differences were detected between spleen cells from TNFα^+/+ ^mice versus TNFα^-/- ^mice regarding IL-6 production upon stimulation with highly purified HMGB1 after 24 hours and 48 hours. Upon stimulation with a suboptimal dose of recombinant HMGB1, however, the splenocytes from TNFα^+/+ ^animals released significantly more IL-6 than cells from the knockout mice (602 ± 112 pg/ml and 304 ± 50 pg/ml, respectively; *P *< 0.05).

**Conclusion:**

Our data show that HMGB1-triggered joint inflammation is not mediated via the TNF pathway. Combined with our previous study, we suggest that HMGB1-triggered arthritis is probably mediated through IL-1 activation.

## Introduction

Rheumatoid arthritis is an autoimmune disease characterized by chronic inflammation in the joints leading to destruction of articular cartilage and bone. The pathogenesis of the disease is complex, involving a wide range of cytokines and endogenous proinflammatory molecules.

High mobility group box chromosomal protein 1 (HMGB1), a nuclear DNA-binding protein, proved recently to be a potent proinflammatory cytokine implicated as an important mediator of arthritis [1]. Increased levels of HMGB1 are found in the joints of rheumatoid arthritis patients [2,3], and the protein triggers arthritis when applied into the joints of naïve mice [4]. HMGB1 is actively released from immune cells in response to different stimuli, including TNFα and lipopolysaccharide (LPS). Previous studies have reported that IFN-γ plays an important role in the regulation of HMGB1 release partly through a TNFα-dependent mechanism [5]. Chen and colleagues demonstrated that direct suppression of TNF activity with neutralizing antibodies or genetic disruption of TNF expression partially attenuated LPS-induced HMGB1 release from macrophages [6]. Once released, HMGB1 generates a positive feedback loop and in turn induces production of several proinflammatory cytokines – such as IL-6, IL-1β and TNFα – by macrophages, thereby sustaining prolonged inflammation [7].

To what extent the ability of HMGB1 to induce arthritis is mediated via the TNFα pathway and whether the presence of TNFα gene affects the proinflammatory cytokine production in response to HMGB1, however, are unknown. In the present study we examined whether the HMGB1-induced joint inflammation is dependent on TNFα signalling.

## Materials and methods

### Mice

Female and male TNFα knockout mice and backcrossed control animals on a C57Bl6 background were bred at the Institute of Immunology, Biomedical Sciences Research Center 'Al. Fleming', Greece. The animals were housed in the animal facility of the Department of Rheumatology and Inflammation Research, University of Göteborg, Sweden. The mice were kept under standard conditions of temperature and light, and were fed laboratory chow and water *ad libitum*. The study was approved by the Ethical Committee of Göteborg University, and the requisitions of the National Board for Laboratory Animals were followed.

### Reagents

Mouse recombinant HMGB1 (rHMGB1) was expressed in *Escherichia coli *and purified to homogeneity as previously described [8]. Preparations were tested for LPS content by the chromogenic *Limulus *amebocyte lysate assay and contained <2 ng endotoxin/μg rHMGB1. Highly purified recombinant endotoxin-free HMGB1 (pHMGB1) (purchased from HMGBiotech, Milano, Italy) was also used for experiments. LPS from *E. coli *serotype 055:B5 was purchased from Sigma (Saint Louis, MO, USA).

### Injection protocol

In the first experiment, TNFα^-/- ^mice and backcrossed control animals on a C57Bl6 background were injected intraarticularly with 5 μg pHMGB1. In the second experiment, mice received the intraarticular injection of 5 μg rHMGB1 and the contralateral knee was injected with 10 ng LPS, which corresponded to the amount of LPS found in rHMGB1 preparations.

### Histologic examination

Three days after the intraarticular injections, the optimal time to trigger synovitis [4], the mice were sacrificed. The knee joints were removed, fixed in 4% formaldehyde, decalcified, embedded in paraffin, sectioned and stained with H&E. All of the slides were assessed in a blinded manner by two researchers (RP and AT). The extent of synovitis was judged on an arbitrary scale from grade 0 to grade 3, as described elsewhere [4].

### *In vitro *experiments

Spleen cells from TNFα^-/- ^mice and from control mice were prepared as previously described [9] and were stimulated with different doses of rHMGB1 and pHMGB1. The corresponding amount of LPS from *E. coli *was used for cell stimulation as a control for rHMGB1. Cell culture supernatants were collected after 24 hours and 48 hours for determination of the IL-6 level, as previously described [9].

For the *in vitro *proliferation assay, splenocyte cultures were prepared; the cells were then incubated for 72 hours in 96-well plates with final concentrations of 0.05 μg/ml, 0.5 μg/ml, and 5 μg/ml pHMGB1. Culture medium was used as a negative control, and concavalin A at a concentration of 2.5 μg/ml as a positive control. The cultures were pulsed with 1 μCi tritiated thymidine 12 hours before harvesting, and the tritiated thymidine uptake was counted in a beta counter. The proliferative response is expressed as the mean ± standard error of the mean (median) counts per minute of triplicate samples from five spleens in each group.

### Statistical analysis

Nonparametric methods were used for statistical comparisons since the data showed a non-normal distribution. Statistical differences between independent groups were calculated using the Mann–Whitney U test. *P *< 0.05 was considered significant.

## Results

### Induction of arthritis by HMGB1 in TNFα^-/- ^mice and in control mice

**Figure 1:**
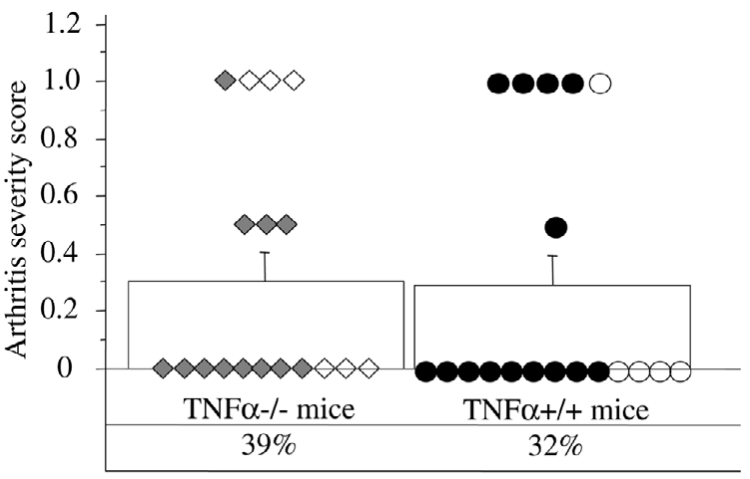
Arthritis induction by high mobility group box chromosomal protein 1 in TNFα^-/- ^mice and controls. Arthritis incidence (%) and arthritis severity scores (mean ± standard error of the mean) are shown in TNFα^-/- ^mice (squares) and in control mice (circles) (*n *= 18 to 19 per group) following a single intraarticular injection of 5 μg high mobility group box chromosomal protein 1 (HMGB1). Empty squares/circles, individual mice (*n* = five to six per group) receiving recombinant HMGB1 containing a minimal amount of lipopolysaccharide (see Materials and methods); filled squares/circles, mice (*n* = 12 to 14 per group) receiving lipopolysaccharide-free HMGB1.

To assess the importance of the TNFα signalling pathway in HMGB1-triggered arthritis, TNFα^-/- ^mice and control TNFα^+/+ ^mice were given a single intraarticular injection of 5 μg pHMGB1 into a knee joint. This dose has been established in previous experiments to induce arthritis [4]. Intraarticular injection of HMGB1 into healthy mouse joints resulted in an overall frequency of 32% to 39% arthritic animals as assessed histologically. No significant differences were found with respect to the severity and incidence of arthritis between mice deficient for TNFα (seven out of 18 mice with arthritis) in comparison with control TNFα^+/+ ^animals (six out of 19). The inflammation was characterized by mild synovitis (Figure 1).

Six mice in both groups received 5 μg rHMGB1 intraarticularly, which is known to contain a higher concentration of LPS. As a control, the contralateral knee joint of those mice was injected with 10 ng LPS, a dose corresponding to the LPS present in the rHMGB1 preparations used for injection. We could not observe any significant differences regarding arthritis severity in mice receiving rHMGB1 between mice with or mice without the functional TNFα gene (Figure 1). Two mice out of the six injected with 10 ng LPS displayed mild synovitis in both groups irrespective of the presence or absence of the TNFα gene. These results indicate that HMGB1 induces joint inflammation *in vivo *independently of the TNFα pathway.

### Cytokine response to HMGB1 in TNF^-/- ^mice

We next examined whether TNFα deficiency affects a proinflammatory cytokine response to HMGB1 *in vitro*. Mouse splenocytes from TNFα^-/- ^mice and from control TNFα^+/+ ^mice were stimulated with 0.05 μg, 0.5 μg and 5 μg pHMGB1 for 24 hours and 48 hours, and the IL-6 production in cell culture supernatants was determined. Stimulation with pHMGB1 did not induce significant IL-6 release from TNFα^-/- ^cells as compared with TNFα^+/+ ^splenocytes at any time point and irrespective of the pHMGB1 concentration used (Table 1).

**Table 1:** Production of IL-6 following 24 hours and 48 hours of stimulation with different doses of purified recombinant endotoxin-free high mobility group box chromosomal protein 1 (pHMGB1) and mouse recombinant high mobility group box chromosomal protein 1 (rHMGB1)

Time	Mice	IL-6 production (pg/ml)				Lipopolysaccharide^a^
			
		0 μg/ml HMGB1	0.05 μg/ml HMGB1	0.5 μg/ml HMGB1	5 μg/ml HMGB1	
PHMGB1						
24 hours	TNFα^-/-^	17.0 ± 2.3	17.6 ± 2.2	17.8 ± 2.4	13.1 ± 4.6	2,460 ± 226
	TNFα^+/+^	16.2 ± 1.9	15.9 ± 2.0	16.8 ± 3.2	7.3 ± 3.1	6,905 ± 1,763
48 hours	TNFα^-/-^	19.1 ± 3.0	20.1 ± 2.9	17.0 ± 1.6	26.3 ± 3.8	
	TNFα^+/+^	19.8 ± 2.2	18.3 ± 2.2	14.2 ± 2.3	20.2 ± 3.2	
RHMGB1						
24 hours	TNFα^-/-^	28 ± 16		304 ± 50* (314)	2,317 ± 264 (2,271)	146 ± 46* (130)
	TNFα^+/+^	68 ± 11		602 ± 112 (558)	3,046 ± 757 (2,504)	344 ± 63 (359)
48 hours	TNFα^-/-^	213 ± 58		468 ± 56* (500)	2,541 ± 258 (2,588)	238 ± 79 (207)
	TNFα^+/+^	263 ± 33		1,133 ± 202 (1,062)	4,114 ± 941 (3,527)	438 ± 62 (408)

Data presented as the mean ± standard error of the mean of 10 mice per each group (pHMGB1), and the mean ± standard error of the mean (median) of four mice in each group (rHMGB1). HMGB1, high mobility group box chromosomal protein 1. ^a^pHMGB1, 5 μg/ml; rHMGB1, 10 ng/ml. *P < 0.05 TNFα^-/- ^mice versus control mice.

In another experiment, spleen cells from TNF^-/- ^mice and from control mice were prepared and stimulated with 0.5 μg/ml and 5 μg/ml rHMGB1 for 24 hours and 48 hours. As a control, the splenocytes were stimulated with 10 ng LPS, a dose corresponding to that present in the highest HMGB1 preparations. The results show that, upon stimulation with 0.5 μg/ml rHMGB1, the TNFα^+/+ ^cells released significantly more IL-6 than cells from the knockout mice after 24 hours (*P *< 0.05) and 48 hours (*P *< 0.03) (Table 1). No significant differences were detected, however, between spleen cells from TNFα^+/+ ^mice versus TNFα^-/- ^mice regarding IL-6 production upon stimulation with 5 μg/ml rHMGB1 after 24 hours and 48 hours. As a control, stimulation with 10 ng/ml LPS (corresponding to the amount found in 5 μg/ml rHMGB1) induced approximately 100 times less IL-6 than stimulation with 5 μg/ml rHMGB1 in TNFα^+/+ ^cultures as well as TNFα^-/- ^splenocyte cultures after 24 hours and 48 hours (Table 1).

### Proliferation

**Figure 2:**
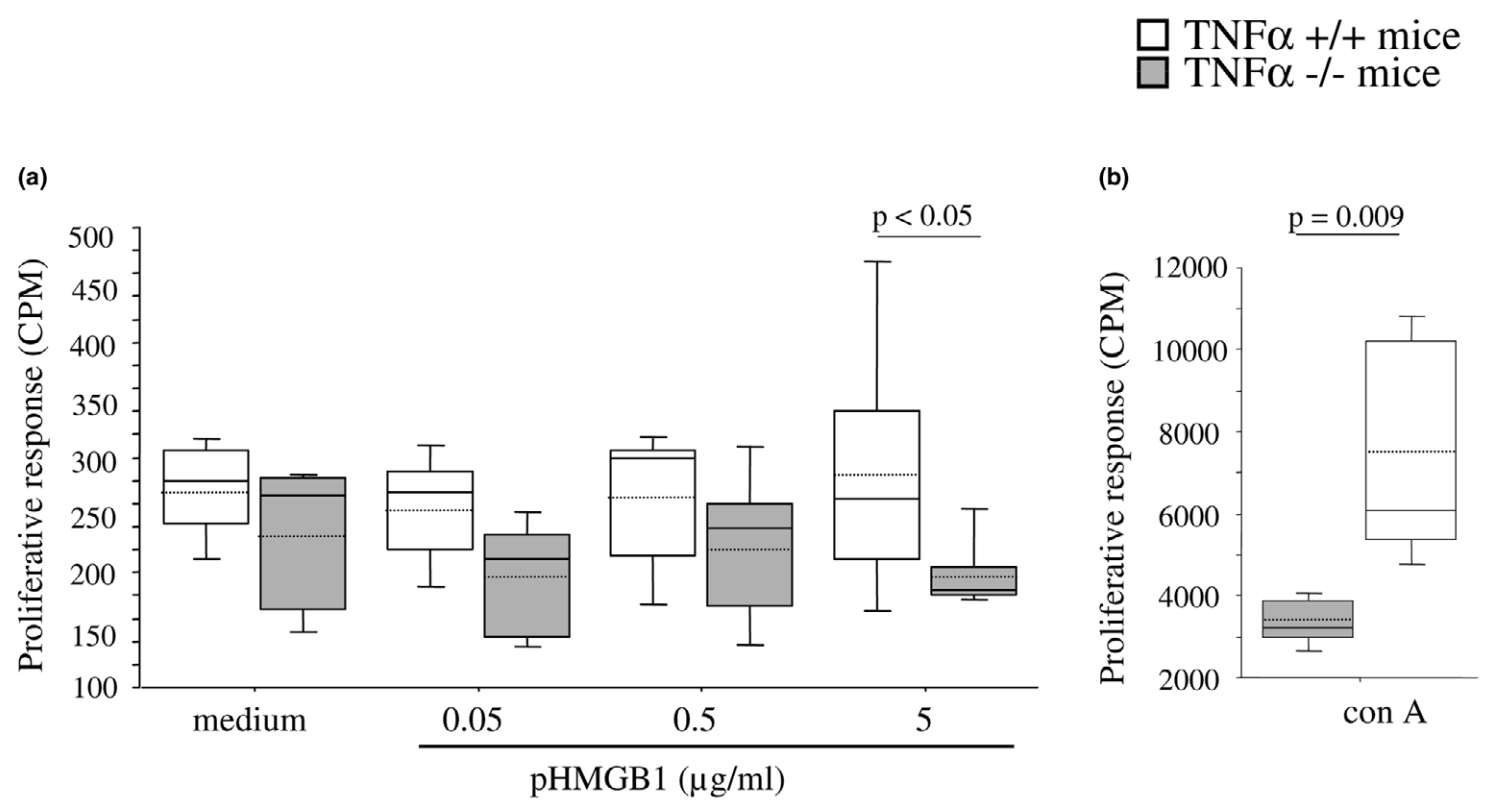
Impact of high mobility group box chromosomal protein 1 on reactivity in the presence/absence of TNFα. Proliferative responses of splenocytes from TNFα^+/+ ^mice and from TNFα^-/- ^mice (*n* = five mice per group) incubated with different doses of **(a) **lipopolysaccharide-free purified recombinant endotoxin-free high mobility group box chromosomal protein 1 (pHMGB1) or **(b) **concavalin A (con A). Box plots, 25th and 75th percentiles; horizontal solid lines, medians; horizontal hatched lines, means; vertical bars, 5th and 95th percentiles. Statistical differences were calculated using the Mann–Whitney U test. CPM, counts per minute.

To assess the impact of HMGB1 on the reactivity of immunocompetent cells in the presence or absence of the TNFα gene, spleen cells from TNFα^+/+ ^mice and from knockout mice were stimulated with different concentrations of endotoxin-free pHMGB1 and the proliferative response was scored after 72 hours. Upon stimulation with the highest HMGB1 dose (5 μg/ml), TNFα^+/+ ^mice had a significantly better proliferative response than their knockout littermates (285 ± 51 (median 264) counts per minute versus 197 ± 15 (median 185) counts per minute, respectively; *P *< 0.05), whereas no differences were seen regarding proliferation at lower HMGB1 concentrations (Figure 2). In addition, knockout mice had a threefold to fourfold lower response to concavalin A, a compound known to act on T lymphocytes, as compared with the TNFα^+/+ ^mice (3,700 ± 246 (median 3,668) counts per minute versus 7,423 ± 1,043 (median 6,550) counts per minute, respectively; *P *= 0.009).

## Discussion

In the present study we demonstrate that HMGB1-triggered joint inflammation is not mediated via the TNF pathway since the arthritis incidence and severity remained similar in mice deficient for TNFα and in backcrossed C57Bl6 control animals.

We have previously shown that the overall frequency and severity of HMGB1-induced arthritis varies between different mouse strains. In the case of C57Bl/6 mice, only 40% develop arthritis – and the severity of inflammation in the joints also proved to be significantly lower in comparison with other mouse strains tested [4]. In the present study we observed mild synovial inflammation in 32% to 39% of mice upon intraarticular injection of HMGB1, which is in accordance with our previous report [4].

In our *in vitro *study we observed no differences regarding IL-6 production from splenocyte cultures between TNFα^-/- ^mice and TNFα^+/+ ^mice in response to stimulation with either endotoxin-free or LPS-containing HMGB1. The latter LPS-containing HMGB1, however, induced a much higher IL-6 response as compared with the endotoxin-free preparation. The higher activity of the recombinant HMGB1 to induce IL-6 release cannot be explained only by the endotoxin contamination since the corresponding dose of LPS found in HMGB1 preparations induced a 100-fold lower production of IL-6 than HMGB1. The synergistic effect of LPS and HMGB1 mediated via Toll-like receptor 4 could account for the increased production of proinflammatory IL-6 release demonstrated in our study.

HMGB1 is a potent inducer of several proinflammatory cytokines. One of these cytokines, IL-1, is considered a crucial mediator in the pathogenesis of destructive arthritis along with TNFα [7]. There is a great deal of crosstalk between IL-1 and TNFα but their pathways differ nevertheless, and production of IL-1 may occur independently of TNFα [10]. In our previous study, we demonstrated that mice deficient for IL-1 receptor did not develop arthritis upon intraarticular administration of HMGB1 [4]. Furthermore, Park and colleagues reported in their study that TNF-receptor associated factor 2 – which is not associated with TLR/IL-1 receptor, but rather with TNF receptors – did not appear to be involved in HMGB1 signalling [11]. Sha and colleagues demonstrated recently that IL-1β was bound to HMGB1 isolated from cells cultured with this cytokine, and addition of anti-IL-1β antibodies or the IL-1 receptor antagonist to cell cultures blocked the proinflammatory activity of HMGB1 [12], further suggesting that the proinflammatory action of HMBG1 is likely to be mediated by IL-1 activation.

## Conclusion

Our results show that pathways other than TNFα are involved in the initiation of joint inflammation in the case of HMGB1-induced arthritis. Combined with our previous study [4], we suggest that HMBG1-triggered arthritis is probably mediated by IL-1 activation.

## Abbreviations

H&E = haematoxylin and eosin; HMGB1 = high mobility group box chromosomal protein 1; IFN = interferon; IL = interleukin; LPS = lipopolysaccharide; pHMGB1 = purified recombinant endotoxin-free high mobility group box chromosomal protein 1; rHMGB1 = mouse recombinant high mobility group box chromosomal protein 1; TNF = tumour necrosis factor.

## Competing interests

The authors declare that they have no competing interests.

## Authors' contributions

RP participated in the design of the study, carried out all the experiments, performed the statistical analysis and drafted the manuscript. I-MJ performed the intraarticular injections and contributed to writing the manuscript. GK provided the TNF knockout/control mice and helped in manuscript preparation. AT conceived of the study, participated in its design and data interpretation, and helped to draft the manuscript. All authors read and approved the final manuscript.
